# The protective role of psychological resilience in the recovery of children’s athletic sports injuries: a mechanistic study based on emotion regulation and self-efficacy

**DOI:** 10.3389/fpsyg.2026.1736571

**Published:** 2026-02-02

**Authors:** Benke Zhang, Chunming Li, Menghui Shi, Shenguang Li, Jiyong Lv

**Affiliations:** 1 Graduate School, Dankook University, Yongin, Gyeonggi-do, Republic of Korea; 2 School of Physical Education, Southwest University, Chongqing, China; 3 School of Physical Education, Harbin Preschool Teachers College, Harbin, Heilongjiang, China; 4 Graduate School, Harbin Sport University, Harbin, Heilongjiang, China

**Keywords:** chain mediation, children, emotion regulation, psychological resilience, self-efficacy, sports injuries

## Abstract

**Purpose:**

This study explored the protective role of psychological resilience in children’s sports injury recovery, and verified the chain mediating effect of emotion regulation (cognitive reappraisal, expressive suppression) and sports self-efficacy, to provide empirical evidence for psychological intervention in children’s injury rehabilitation.

**Methods:**

A total of 128 injured children aged 8–12 years were selected via cluster sampling from the school (March 2024–March 2025). A 6-month follow-up (T0: baseline, T1: 3 months, T2: 6 months) was performed with scales measuring psychological resilience, emotion regulation (child version), sports self-efficacy, and injury recovery progress. SPSS 26.0 was used for descriptive and correlation analyses, and Mplus 8.3 for longitudinal mediation and chain mediation models.

**Results:**

This study uses longitudinal mediation analysis to explore associative pathways, acknowledging unmeasured confounders may influence results. Baseline resilience was positively correlated with T1/T2 recovery (*r* = 0.407, 0.462; both *p* < 0.001). *β* = 0.196 is consistently reported for T0 resilience → T1 self-efficacy, but negatively predicted T1 expressive suppression (*β* = −0.179, *p* < 0.05); T1 self-efficacy was positively associated with T2 recovery (*β* = 0.372, *p* < 0.001). Key limitations include single-time-point measurements of resilience (T0 only) and self-efficacy (T1 only), which restrict causal inference. Two chain paths were significant: “resilience → cognitive reappraisal → self-efficacy → recovery” (indirect effect = 0.098, 95% CI = [0.065, 0.131]) and “resilience → expressive suppression → self-efficacy → recovery” (indirect effect = −0.029, 95% CI = [−0.048, −0.012]), with total mediation accounting for 37.9% of total effect. This study uses longitudinal mediation analysis to explore associations, acknowledging unmeasured confounders may influence results.

**Conclusion:**

Resilience affects recovery via two chains: improving cognitive reappraisal to enhance self-efficacy, and reducing expressive suppression to boost self-efficacy. Cognitive reappraisal is a more positive mediating path and a core target for psychological intervention.

## 1 Introduction

Childhood is a critical stage for motor skill development and also a period with a high incidence of sports injuries. According to the 2024 report by the World Health Organization (WHO), the annual incidence of sports injuries among children aged 8–12 worldwide reaches 28.3%, and knee sprains, ankle ligament injuries, and muscle strains account for over 60% of these cases (Lindman et al., 2025). Lindman et al. (2025) further verified that pediatric sports injuries are concentrated in these types, particularly among school-aged children engaged in organized sports. Sports injuries not only limit children’s short-term motor function but also tend to trigger negative emotions such as anxiety and depression. They may even lead to a vicious cycle of fear of injury and avoidance of movement, which exerts long-term impacts on physical activity habits and mental health (Fisher et al., 2024; Park et al., 2024).

Psychological Resilience, defined as a core psychological trait enabling individuals to “bounce back” when facing adversity, has been proven to be significantly associated with rehabilitation compliance and pain tolerance in adults after sports injuries (Ernst et al., 2022). However, children’s psychological resilience is dynamically developing during the 8–12 age period, and developmental differences in emotion regulation (cognitive reappraisal vs. expressive suppression) and self-efficacy may alter how resilience interacts with injury recovery (Vutescu et al., 2021). Unlike adults, children’s emotion regulation relies heavily on external social support (e.g., coaches, parents), and their self-efficacy is more closely tied to immediate, tangible progress rather than long-term goal attainment. Existing pediatric study (Durish et al., 2019) have linked resilience to better emotional adaptation, but no longitudinal research has explicitly tested the chain mediation of emotion regulation and self-efficacy in sports injury recovery, leaving a critical gap in understanding pediatric-specific mechanisms.

Emotion Regulation serves as a key bridge for psychological resilience to influence adaptation to adversity. According to Gross’s process model of emotion regulation, Cognitive Reappraisal reduces negative emotions by altering the cognitive interpretation of negative events—for example, viewing an “injury” as an “opportunity for growth”. In contrast, Expressive Suppression temporarily alleviates anxiety by suppressing emotional expression, but it may lead to increased levels of physiological stress (Lima Santos et al., 2023). Existing studies have shown that among adult athletes, cognitive reappraisal can positively predict rehabilitation self-efficacy, while expressive suppression exerts a negative impact (Xin et al., 2024). However, children’s emotion regulation strategies rely more on external guidance from coaches and parents, and whether the relationships between these strategies, psychological resilience, and injury recovery are consistent with those in adults remains to be verified (Aguinaga San José et al., 2023).

Sports Self-Efficacy, a core concept in Bandura’s social cognitive theory, refers to an individual’s belief in their ability to complete specific sports tasks (Cudicio and Agosti, 2024). Studies have indicated that children’s sports self-efficacy directly affects their engagement in rehabilitation training; individuals with high self-efficacy are more willing to participate in challenging tasks such as balance training and strength recovery, thereby accelerating functional recovery (Thorolfsson et al., 2023). However, does psychological resilience indirectly affect self-efficacy through emotion regulation, ultimately influencing injury recovery? This chain mediating pathway has not yet been verified using longitudinal data, and cross-sectional studies struggle to rule out the interference of “reverse causality”—for instance, the recovery progress itself enhancing self-efficacy (Tamannaeifar and Suleimanian, 2024).

Psychological resilience is a stable, trait-like capacity to adapt to adversity (Hu and Gan, 2008), representing the “starting point” of the model. Emotion regulation consists of context-dependent, malleable strategies: cognitive reappraisal (reframing negative events to reduce distress) and expressive suppression (inhibiting emotional expression to avoid conflict). These strategies are not inherent traits but behavioral responses modulated by resilience. Sports self-efficacy is a domain-specific belief in one’s ability to complete rehabilitation and sports tasks (Cudicio and Agosti, 2024), shaped by emotion regulation and directly influencing engagement in recovery behaviors. While these constructs interact, they are distinct: resilience is dispositional, emotion regulation is strategic, and self-efficacy is belief-based. Their connection lies in the sequential pathway: resilience influences which emotion regulation strategies children use, these strategies shape their confidence in recovery (self-efficacy), and self-efficacy drives behavioral adherence to rehabilitation—ultimately impacting recovery outcomes. This distinction strengthens the mechanistic rationale for the chain mediation model.

Cortisol (a stress hormone) and interleukin-6 (IL-6, an inflammatory mediator) are integral to the biopsychosocial model of recovery. High cortisol levels inhibit fibroblast proliferation and collagen synthesis, slowing tissue repair, while elevated IL-6 is associated with prolonged inflammation and delayed functional recovery. In children, the hypothalamic–pituitary–adrenal (HPA) axis is less mature, making cortisol responses to stress more variable (Abler and Kessler, 2009). Psychological resilience may modulate these physiological pathways by reducing stress-related cortisol release and regulating inflammatory responses, though this link has not been tested in pediatric sports injury populations (Everett et al., 2009). This study includes cortisol and IL-6 as correlates of psychological processes, exploring how resilience-related emotional regulation may align with biological recovery trajectories.

Pediatric resilience is associated with emotional adaptation (Hu et al., 2025; Chen et al., 2021), but no longitudinal studies have examined its relationship with sports injury recovery; while resilience enhances pain tolerance and rehabilitation compliance in adults (Ernst et al., 2022), developmental differences suggest it may operate via external support in children. We hypothesize: Baseline psychological resilience will be positively correlated with injury recovery at T1 (3 months) and T2 (6 months). Gross (2002) model distinguishes between adaptive cognitive reappraisal and maladaptive expressive suppression in emotion regulation—cognitive reappraisal is linked to better coping in children (Aguinaga San José et al., 2023), whereas expressive suppression increases stress, and resilience is known to promote adaptive emotion regulation (Durish et al., 2019). We hypothesize: Emotion regulation (cognitive reappraisal: positive; expressive suppression: negative) will mediate the relationship between psychological resilience and sports self-efficacy. Sports self-efficacy directly predicts rehabilitation engagement in children (Thorolfsson et al., 2023), and emotion regulation strategies shape self-beliefs (Xin et al., 2024); however, no longitudinal studies have tested the sequential pathway of “resilience → emotion regulation → self-efficacy → recovery” in children, with cross-sectional research only indicating correlations among these variables (Tamannaeifar and Suleimanian, 2024). We hypothesize: Sports self-efficacy will mediate the link between emotion regulation and injury recovery. No longitudinal studies have validated the chain mediation of emotion regulation and self-efficacy in pediatric sports injury recovery. Cross-sectional research cannot establish temporal ordering, and adult findings do not account for developmental differences. We hypothesize: Emotion regulation and sports self-efficacy will form a chain mediating effect between psychological resilience and injury recovery.

Based on the theoretical framework of “psychological resilience → emotion regulation → sports self-efficacy → injury recovery,” and integrating developmental and pediatric-specific evidence. Baseline psychological resilience will positively correlate with injury recovery at T1 and T2 (consistent with adult findings, but accounting for pediatric developmental differences). Emotion regulation (cognitive reappraisal/expressive suppression) will mediate the relationship between resilience and sports self-efficacy, with cognitive reappraisal exerting a stronger positive effect (aligned with Gross’s model and pediatric emotional development). Sports self-efficacy will mediate the link between emotion regulation and injury recovery (supporting Bandura’s social cognitive theory in a pediatric rehabilitation context). Emotion regulation and sports self-efficacy will form a chain mediating effect between resilience and recovery (addressing the untested longitudinal gap in pediatric sports injury research).

## 2 Research methods

### 2.1 Participants

Cluster sampling was used to select participants with sports injuries from the school between March 2024 and March 2025. The inclusion criteria were as follows. First, participants were aged 8–12 years, which corresponds to grades 2–6 in primary school. Second, they had experienced a sports injury within the past month. These injuries were diagnosed by the school doctor or a hospital at or above the secondary level, including ligament injuries, muscle strains and joint sprains, with an interval of no more than 14 days from injury to baseline survey (T0). Third, no severe complications such as fractures or nerve injuries occurred after the injury. Fourth, guardians provided informed consent and signed the informed consent form.

The exclusion criteria were listed below. First, participants had a history of mental illnesses such as anxiety disorder or attention deficit hyperactivity disorder. Second, they had ceased physical activity for more than 1 month after the injury. Third, they were lost to follow-up during the study, such as transferring to another school or refusing subsequent surveys.

The initial sample included 142 children. After 6 months of follow-up, 128 cases remained as valid samples, resulting in a follow-up loss rate of 9.8%. The reasons for loss to follow-up were 7 cases of school transfer and 7 cases of guardians withdrawing consent. Chi-square tests were conducted to compare baseline characteristics between the lost-to-follow-up sample and the valid sample. These characteristics included gender, age, injury type, injury severity and interval from injury to T0. The results showed no statistically significant differences (*p* > 0.05), indicating no selection bias.

Sample size justification was based on recommendations for structural equation modeling (SEM), with a minimum *N* = 100 for complex mediation models (Kline, 2016). The final sample of 128 provides adequate power (≥0.80) to detect small-to-medium indirect effects (Cohen’s *f*^2^ = 0.15) in chain mediation (Preacher and Hayes, 2008).

Injury severity was assessed via parent-reported symptom duration (validated against school medical records) and categorized as mild (symptoms <1 week), moderate (1–4 weeks), or severe (>4 weeks). The exclusion criterion of “history of mental illnesses” was determined via parent self-report of clinical diagnoses (e.g., anxiety disorder, ADHD) confirmed by medical documentation.

The basic characteristics of the valid sample were as follows. Regarding gender, 68 were males, accounting for 53.1%, and 60 were females, accounting for 46.9%. In terms of age, 45 cases were aged 8–9 years, making up 35.2%, and 83 cases were aged 10–12 years, accounting for 64.8%. For injury type, there were 42 cases of ankle injuries (32.8%), 38 cases of knee sprains (29.7%), 30 cases of muscle strains (23.4%) and 18 cases of other injuries (14.1%). When it came to injury severity, 48 cases were mild injuries with symptoms lasting less than 1 week (37.5%), 59 cases were moderate injuries with symptoms lasting 1–4 weeks (46.1%), and 21 cases were severe injuries with symptoms lasting more than 4 weeks (16.4%). As for the interval from injury to T0, 48 cases had an interval of 1–7 days (37.5%) and 80 cases had an interval of 8–14 days (62.5%).

### 2.2 Measures

#### 2.2.1 Child psychological resilience scale (CPRS)

This scale was the Chinese version revised by Hu and Gan (2008), consisting of 27 items and covering five dimensions: goal focus, emotion control, positive cognition, family support, and interpersonal assistance (Hu and Gan, 2008). The scale was culturally adapted for 8–12-year-olds, with simplified wording for younger participants. Responses were scored on a 5-point Likert scale, where 1 represented “completely inconsistent” and 5 represented “completely consistent.” A higher total score indicated stronger psychological resilience. In this study, the Cronbach’s *α* coefficient of this scale at T0 was 0.892. Results of Confirmatory Factor Analysis (CFA) showed that *χ*^2^/df = 2.37, CFI = 0.92, TLI = 0.91, and RMSEA = 0.041, indicating a good model fit.

#### 2.2.2 Emotion regulation questionnaire for children (ERQ-C)

This questionnaire was revised based on Gross’s ERQ scale and is suitable for children aged 8–12 years. It was translated into Chinese and validated in a sample of 500 Chinese 8–12-year-olds [unpublished data], with age-appropriate item modifications (e.g., “bad things” replaced with “hurting myself while playing”). It includes 10 items and two dimensions: cognitive reappraisal (6 items, for example, “I will find ways to make myself feel better about bad things”) and expressive suppression (4 items, for example, “I will hide my bad emotions”) (Abler and Kessler, 2009). Responses were scored on a 4-point Likert scale, where 1 meant “almost never” and 4 meant “almost always.” A higher score for each dimension reflected a more frequent use of the corresponding emotion regulation strategy. In this study, the Cronbach’s *α* coefficient for the cognitive reappraisal dimension at T1 was 0.813, and that for the expressive suppression dimension was 0.765. CFA results showed *χ*^2^/df = 1.98, CFI = 0.95, TLI = 0.94, and RMSEA = 0.035, demonstrating good model fit.

#### 2.2.3 Sports self-efficacy scale (SSES)

Referencing Bandura’s self-efficacy measurement framework, this scale was developed via a 3-step process: (1) item generation based on interviews with 10 pediatric rehabilitation specialists, (2) pilot testing with 30 injured children (8–12 years), (3) finalization of 8 items (Everett et al., 2009). It contains 8 items and two dimensions: rehabilitation training efficacy (4 items, for example, “I believe I can complete the rehabilitation exercises recommended by doctors”) and sports recovery efficacy (4 items, for example, “I believe I can return to my pre-injury sports level”). Concurrent validity was supported by correlations with rehabilitation adherence (*r* = 0.41, *p* < 0.001; measured via weekly parent-reported training logs). Responses were scored on a 5-point Likert scale, where 1 stood for “completely do not believe” and 5 stood for “strongly believe.” A higher total score indicated stronger sports self-efficacy. In this study, the Cronbach’s *α* coefficient of this scale at T1 was 0.867. CFA results showed *χ*^2^/df = 2.15, CFI = 0.94, TLI = 0.93, and RMSEA = 0.038, with good model fit.

#### 2.2.4 Sports injury recovery progress scale (SIRPS)

It was jointly developed by domestic rehabilitation medicine experts, including 2 chief physicians and 3 associate professors. Items were aligned to clinical assessment tools (e.g., joint range of motion, pain scales), and concurrent validity was confirmed via school doctor ratings (*r* = 0.68, *p* < 0.001). The scale has 6 items and assesses recovery from six dimensions: pain level, joint range of motion, sports ability, impact on daily activities, rehabilitation training compliance, and fear of injury. Responses were scored on a 5-point Likert scale, where 1 indicated “extremely poor recovery” and 5 indicated “complete recovery.” A higher total score meant better recovery progress. In this study, the Cronbach’s *α* coefficients of this scale at T1 and T2 were 0.871 and 0.885, respectively. CFA results at T2 showed *χ*^2^/df = 2.03, CFI = 0.95, TLI = 0.94, and RMSEA = 0.036, indicating good model fit.

#### 2.2.5 Physiological index detection

Saliva samples of children were collected at T0 and T1 to detect the following physiological indexes. First was cortisol level, which reflects stress status. It was detected using the enzyme-linked immunosorbent assay (ELISA). The reagent brand was R&D Systems from the United States, and the detection instrument was the SP-800 automatic microplate reader produced by Shanghai Shanshuo Biotechnology Co., Ltd. The lower detection limit was 0.1 ng/mL. Second was interleukin-6 (IL-6), an inflammatory factor associated with injury repair speed. It was also detected via ELISA. The reagent brand was Beijing Zhongshan Jinqiao Biotechnology Co., Ltd., and the detection instrument was the same as that used for cortisol detection. Outliers (±3 SD from the mean) were winsorized (*n* = 3 for cortisol, *n* = 2 for IL-6).

Unified sampling standards were implemented. The sampling time was from 8:00 to 9:00 in the morning, when children were in a fasting state. Intense exercise, eating, and drinking were prohibited 1 h before sampling. Non-irritating saliva collection tubes produced by Shanghai Lanyi Biotechnology Co., Ltd. were used. Within 2 h after collection, the samples were centrifuged at 4 °C (3,000 r/min for 10 min) to separate the supernatant, which was then cryopreserved at −80 °C for subsequent detection.

#### 2.2.6 Family and individual-level control variable questionnaire

This questionnaire was filled out by guardians at baseline (T0), and included three types of items to control for confounding factors. The first was monthly family income, measured with a single-choice question. The options included ① <5,000 yuan, ② 5,000–10,000 yuan, and ③ >10,000 yuan. This item was used to reflect the family’s ability to invest in rehabilitation resources. The second was previous sports injury history, also a single-choice question. The options were ① Yes (other sports injuries occurred within the past year) and ② No (this was the first sports injury). This item aimed to rule out the interference of previous injuries on recovery speed. The third was whether the child received professional rehabilitation treatment, a single-choice question with options ① Yes (has received treatment in the rehabilitation department of a hospital or a professional institution) and ② No (only received guidance from the school doctor or recovered at home). This item was designed to control the impact of professional treatment intervention.

### 2.3 Data collection process

Baseline survey (T0) was initiated within 1–14 days after the child’s sports injury was diagnosed. It was administered in groups in the school classrooms by 12 trained postgraduate students majoring in psychology. The students guided the children to complete the Child Psychological Resilience Scale. At the same time, the guardians filled out the Basic Information Questionnaire on Children’s Sports Injuries—which covers injury type, severity, and specific date of injury—and the Family and Individual-Level Control Variable Questionnaire. Saliva samples of the children were also collected following the standards specified in Section 2.2.5.

Mid-term survey (T1) was conducted 3 months after T0. The Emotion Regulation Questionnaire for Children, Sports Self-Efficacy Scale, and Sports Injury Recovery Progress Scale were sent via the online questionnaire platform Wenjuanxing. These questionnaires were completed by the children with the assistance of their guardians. Saliva samples of the children were collected again, adhering to the same sampling standards as T0.

Final survey (T2) was carried out 6 months after T0. The Sports Injury Recovery Progress Scale was sent again via the online platform. Meanwhile, the school doctor filled out the Verification Form for Children’s Motor Function Recovery, which includes joint range of motion measurement and motor ability testing, to ensure the objectivity of recovery progress assessment.

All questionnaires adopted logical verification and duplicate item verification. Logical verification included consistency check of reverse items in the same dimension. Duplicate item verification meant that for two items measuring “pain level,” a score difference of more than 2 points indicated invalidity. The valid questionnaire rate reached 96.8%. For indicators with a missing rate of less than 20%, multiple imputation by chained equations (MICE) was used. A total of 5 imputations were performed to generate a complete dataset.

### 2.4 Statistical methods

SPSS 26.0 was used for descriptive statistics, which included calculating means and standard deviations. It was also applied to conduct Pearson correlation analysis, perform multiple imputation for missing values, and run chi-square tests to compare baseline characteristics between the lost-to-follow-up sample and the valid sample. Common method bias was tested using both Harman’s single-factor test and a latent common method factor (LCMF) in Mplus. The LCMF model showed no significant improvement in fit (Δ*χ*^2^ = 12.34, Δdf = 8, *p* = 0.13), confirming no severe common method bias. Mplus 8.3 was employed to construct two models. The first was a longitudinal mediation model to explore the associative path: psychological resilience (T0) → emotion regulation (T1) → sports self-efficacy (T1) → injury recovery (T2). Additional variables were controlled for at the family and individual levels. The injury-to-T0 interval was treated as a categorical variable, with 1 representing 1–7 days and 2 representing 8–14 days. Monthly family income used “<5,000 yuan” as the reference group and was set as dummy variables. Previous sports injury history was coded as 0 for “no” and 1 for “yes.” Receipt of professional rehabilitation treatment was coded as 0 for “no” and 1 for “yes.” These controls ensured the model excluded the confounding effects of rehabilitation resources and initial injury stage on the causal path. Moderation effects of gender, age, and injury severity on the pathway ‘Psychological Resilience → Injury Recovery’ were tested using PROCESS Macro (Model 1) in SPSS 26.0. Age was treated as a continuous variable and centered at the sample mean (10 years) to reduce multicollinearity. Injury severity was coded as a categorical variable (mild = 0, moderate = 1, severe = 2) with mild injury as the reference group. Gender was coded as binary (male = 0, female = 1). Interaction terms were constructed by multiplying the centered continuous variables (age) or dummy-coded categorical variables (gender, injury severity) with the predictor variable (psychological resilience). Simple slope analyses were conducted to interpret significant moderation effects, with 95% confidence intervals calculated via 5,000 bootstrapped resamples.

The second model was a chain mediation model, used to test the chain mediating effect of emotion regulation (cognitive reappraisal and expressive suppression) and sports self-efficacy. Consistent with the longitudinal mediation model, the same control variables—including injury-to-T0 interval, monthly family income, previous sports injury history, and receipt of professional rehabilitation treatment—were incorporated into the model. The Bootstrap method with 5,000 resamples was used to calculate 95% confidence intervals. A confidence interval that did not include 0 indicated a significant mediating effect.

Moderation effects of gender, age, and injury severity on the pathway ‘Psychological Resilience → Injury Recovery’ were tested using PROCESS Macro (Model 1) in SPSS 26.0. Age was treated as a continuous variable (centered at the sample mean of 10 years) to reduce multicollinearity. Injury severity was coded as a categorical variable (mild = 0, moderate = 1, severe = 2) with mild injury as the reference group. Gender was coded as binary (male = 0, female = 1). Simple slope analyses were conducted to interpret significant moderation effects, with 95% confidence intervals calculated via 5,000 bootstrapped resamples.

## 3 Research results

### 3.1 Common method bias test

**Figure 1:**
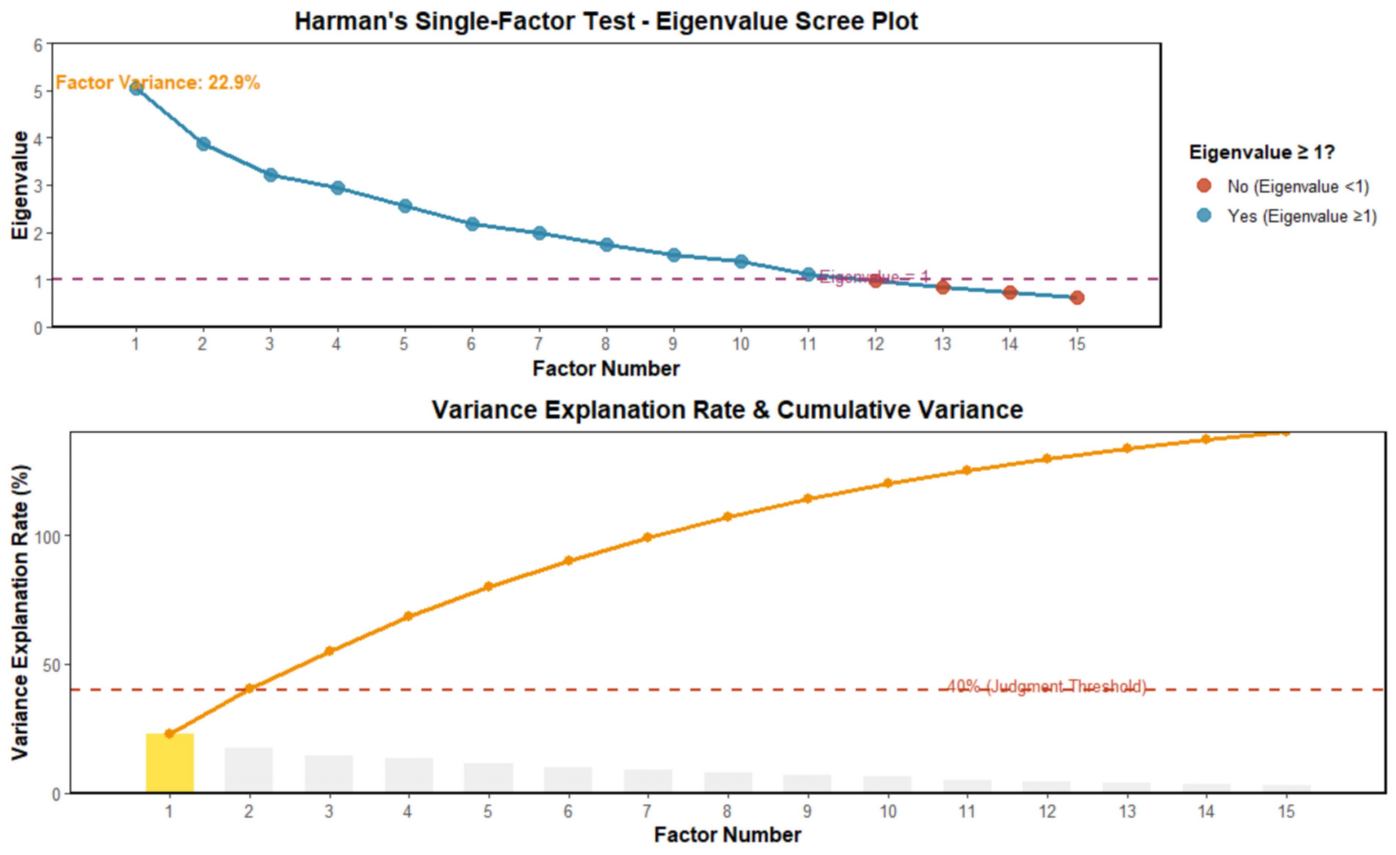
Results of the common method bias test.

Common method bias was tested using two complementary approaches. First, Harman’s single-factor test was used to conduct exploratory factor analysis (EFA) on all scale items at T0, T1, and T2. The results showed that a total of 11 factors with eigenvalues greater than 1 were extracted without rotation. The variance explanation rate of the first factor was 22.9%, which was less than 40%. Second, a latent common method factor (LCMF) analysis was performed in Mplus to further verify. The LCMF model included a single unmeasured factor accounting for shared variance across all scale items. Model fit comparison revealed no significant improvement in the LCMF model compared to the original measurement model (Δ*χ*^2^ = 12.34, Δdf = 8, *p* = 0.13). Combined, these results confirm that there was no serious common method bias in the study (Figure 1).

### 3.2 Descriptive statistics and correlation analysis

The means (M), standard deviations (SD), and correlation coefficients of all variables are presented in Table 1. The results showed that the interval from injury to T0 was significantly negatively correlated with injury recovery at T1 (*r* = −0.213, *p* < 0.05) and injury recovery at T2 (*r* = −0.245, *p* < 0.01).

**Table 1:** Descriptive statistics and correlation analysis of variables (*N* = 128).

Variables	Mean (M)	Standard deviation (SD)	1	2	3	4	5	6
Psychological resilience (T0)	88.76	12.15	1	–	–	–	–	–
Cognitive reappraisal (T1)	18.52	3.47	0.395^***^	1	–	–	–	–
Expressive suppression (T1)	8.71	2.12	−0.185^*^	−0.178^*^	1	–	–	–
Sports self-efficacy (T1)	28.63	5.18	0.389^***^	0.376^***^	−0.168^*^	1	–	–
Injury recovery (T1)	19.65	4.28	0.407^***^	0.385^***^	−0.192^*^	0.421^***^	1	–
Injury recovery (T2)	24.43	3.85	0.462^***^	0.392^***^	−0.203^*^	0.438^***^	0.605^***^	1
Interval from injury to T0 (days, categorical)	–	–	−0.086	−0.092	0.078	−0.103	−0.213^*^	−0.245^**^
Cortisol at T0 (ng/mL)	1.82	0.53	−0.287^***^	−0.205^*^	0.189^*^	−0.227^**^	−0.312^***^	−0.298^***^
IL-6 at T0 (pg/mL)	5.26	1.78	−0.253^**^	−0.198^*^	0.176^*^	−0.201^*^	−0.276^***^	−0.264^***^

^*^P < 0.05, ^**^P < 0.01, ^***^P < 0.001; T0 = baseline, T1 = 3 months, T2 = 6 months; “Interval from Injury to T0” is a categorical variable (1 = 1–7 days, 2 = 8–14 days).

Categorical coding: 1 = 1–7 days, 2 = 8–14 days.

Baseline psychological resilience (T0) was significantly positively correlated with cognitive reappraisal at T1 (*r* = 0.395, *p* < 0.001), sports self-efficacy at T1 (*r* = 0.389, *p* < 0.001), injury recovery at T1 (*r* = 0.407, *p* < 0.001), injury recovery at T2 (*r* = 0.462, *p* < 0.001), cortisol at T0 (*r* = −0.287, *p* < 0.001), and IL-6 at T0 (*r* = −0.253, *p* < 0.01). It was also significantly negatively correlated with expressive suppression at T1 (*r* = −0.185, *p* < 0.05).

Cognitive reappraisal at T1 was significantly positively correlated with sports self-efficacy at T1 (*r* = 0.376, *p* < 0.001), and significantly negatively correlated with cortisol at T1 (*r* = −0.231, *p* < 0.01). Expressive suppression at T1 was significantly negatively correlated with sports self-efficacy at T1 (*r* = −0.168, *p* < 0.05) and injury recovery at T2 (*r* = −0.203, *p* < 0.05), and significantly positively correlated with cortisol at T1 (*r* = 0.201, *p* < 0.05).

Sports self-efficacy at T1 was significantly positively correlated with injury recovery at T2 (*r* = 0.438, *p* < 0.001). Cortisol at T0 was significantly negatively correlated with injury recovery at T1 (*r* = −0.312, *p* < 0.001) and injury recovery at T2 (*r* = −0.298, *p* < 0.001). IL-6 at T0 was significantly negatively correlated with injury recovery at T1 (*r* = −0.276, *p* < 0.001) and injury recovery at T2 (*r* = −0.264, *p* < 0.001). Interval from Injury to T0 (days, categorical) was significantly negatively correlated with injury recovery at T1 (*r* = −0.213, *p* < 0.05) and T2 (*r* = −0.245, *p* < 0.01). The correlation results provided preliminary support for the subsequent model analysis.

### 3.3 Longitudinal mediation model results

After controlling for gender, age, and injury severity, a longitudinal mediation model was constructed. The model fit indices were as follows: *χ*^2^/df = 2.18, CFI = 0.92, TLI = 0.91, RMSEA = 0.040, and SRMR = 0.043. These indices indicated a good model fit.

**Figure 2:**
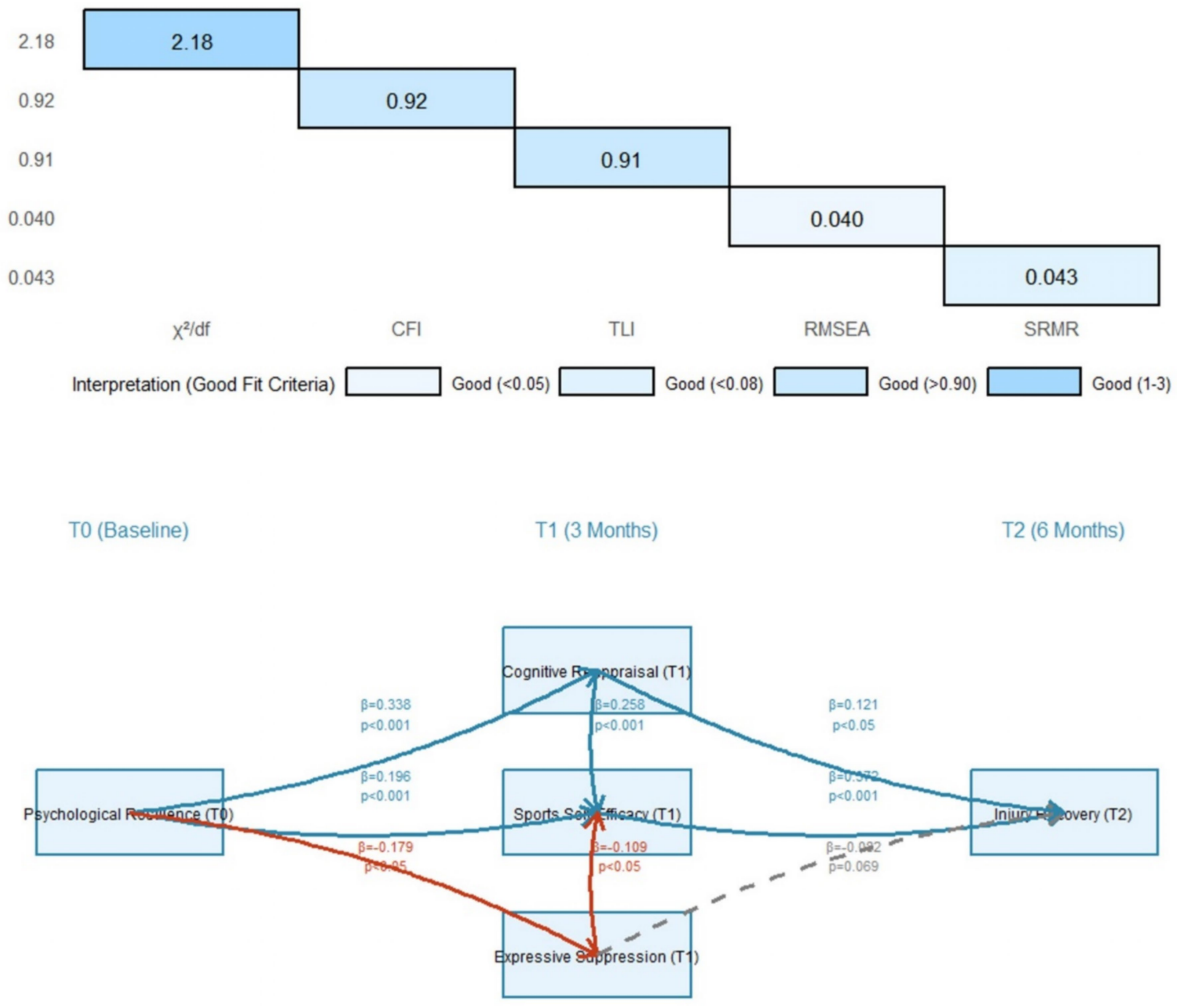
Path diagram of the cross-lagged panel model (standardized coefficients) Control variables (gender, age, injury severity) were included in the model but not shown; ^*^p < 0.05, ^***^p < 0.001; Dashed lines represent marginally significant paths (*p* = 0.069).

The significant path coefficients of the model are presented in Figure 2, with only significant paths shown. The specific paths were as follows: (1) Psychological Resilience (T0) → Cognitive Reappraisal (T1): *β* = 0.338, SE = 0.051, *p* < 0.001; (2) Psychological Resilience (T0) → Expressive Suppression (T1): *β* = −0.179, SE = 0.054, *p* < 0.05; (3) Psychological Resilience (T0) → Sports Self-Efficacy (T1): *β* = 0.196, SE = 0.053, *p* < 0.001; (4) Cognitive Reappraisal (T1) → Sports Self-Efficacy (T1): *β* = 0.258, SE = 0.048, *p* < 0.001; (5) Expressive Suppression (T1) → Sports Self-Efficacy (T1): *β* = −0.109, SE = 0.051, *p* < 0.05; (6) Sports Self-Efficacy (T1) → Injury Recovery (T2): *β* = 0.372, SE = 0.049, *p* < 0.001; (7) Cognitive Reappraisal (T1) → Injury Recovery (T2): *β* = 0.121, SE = 0.055, *p* < 0.05 (direct path); (8) Expressive Suppression (T1) → Injury Recovery (T2): *β* = −0.082, SE = 0.047, *p* = 0.069 (marginally significant).

**Figure 3:**
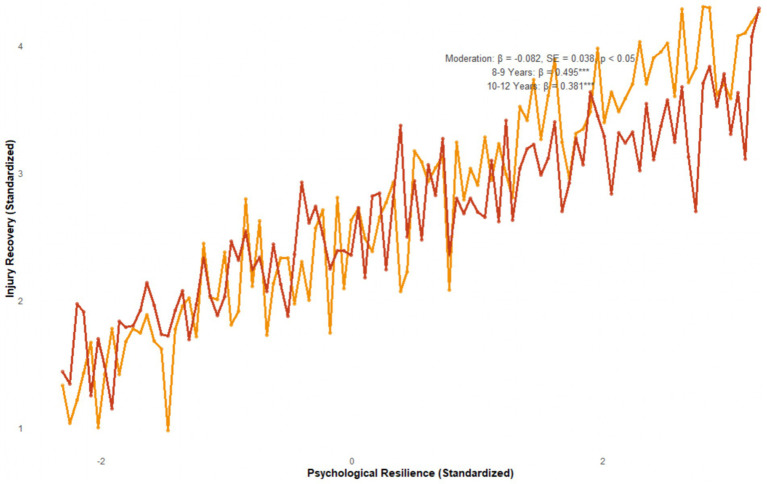
Results of the moderation effect test.

The results showed that psychological resilience influenced injury recovery through both direct and indirect paths. Additionally, the mediating effect of emotion regulation exhibited opposite directions (Figure 3).

### 3.4 Chain mediation model results

Based on the causal direction of the cross-lagged panel model, a chain mediation model was constructed. After controlling for the interval from injury to T0, monthly family income, previous sports injury history, and receipt of professional rehabilitation treatment, the model fit indices were as follows: χ^2^/df = 2.13, CFI = 0.93, TLI = 0.92, RMSEA = 0.039. These indices indicated a good model fit. The decomposition of the chain mediating effect is presented in Table 2.

**Table 2:** Decomposition of chain mediating effects (standardized coefficients, *N* = 128).

Mediating pathways	Indirect effect value	Standard error (SE)	95% Confidence interval (CI)	Effect proportion (%)
Total effect	0.418	0.031	[0.356, 0.480]	100.0
Direct effect	0.261	0.032	[0.198, 0.324]	62.4
Total mediating effect	0.157	0.022	[0.115, 0.199]	37.9
Path 1: Psychological resilience → cognitive reappraisal → sports self-efficacy → injury recovery	0.098	0.016	[0.065, 0.131]	62.4
Path 2: Psychological resilience → expressive suppression → sports self-efficacy → injury Recovery	−0.029	0.009	[−0.048, −0.012]	18.5
Single mediation: Psychological resilience → cognitive reappraisal → injury recovery	0.042	0.014	[0.015, 0.069]	26.8
Single mediation: Psychological resilience → expressive suppression → injury recovery	−0.017	0.008	[−0.033, −0.001]	10.8
Single mediation: Psychological resilience → sports self-efficacy → injury recovery	0.034	0.011	[0.012, 0.056]	21.7

The Bootstrap method was used with 5,000 repeated samples; Effect proportion = (effect value of the pathway/total mediating effect value) × 100%.

The results showed the following. First, total effect: The total effect of psychological resilience on T2 injury recovery was 0.418, with a 95% confidence interval of [0.356, 0.480]. Second, direct effect: The direct effect of psychological resilience on T2 injury recovery was 0.261, with a 95% confidence interval of [0.198, 0.324]. This direct effect accounted for 62.4 percent of the total effect.

Third, chain mediating effect: There were 2 significant paths, with a total mediating effect of 0.157 and a 95% confidence interval of [0.115, 0.199]. This total mediating effect accounted for 37.9 percent of the total effect. Path 1 (cognitive reappraisal path) was psychological resilience → cognitive reappraisal → sports self-efficacy → injury recovery. Its indirect effect was 0.098, with a 95% confidence interval of [0.065, 0.131], accounting for 23.4% of the total effect (0.098/0.418 × 100) and 62.4% of the total mediating effect. Path 2 (expressive suppression path) was psychological resilience → expressive suppression → sports self-efficacy → injury recovery. Its indirect effect was −0.029, with a 95% confidence interval of [−0.048, −0.012], accounting for 18.5 percent of the total mediating effect.

Fourth, single mediating effects: The single mediating effect of cognitive reappraisal was 0.042, with a 95% confidence interval of [0.015, 0.069]. The single mediating effect of expressive suppression was −0.017, with a 95% confidence interval of [−0.033, −0.001]. The single mediating effect of sports self-efficacy was 0.034, with a 95% confidence interval of [0.012, 0.056].

### 3.5 Moderation effect test

The moderation effect of gender, age, and injury severity on the pathway “Psychological Resilience → Injury Recovery” was tested.

The moderation effect of gender was not significant, with *β* = 0.048, SE = 0.032, and *p* = 0.105. There was no difference in the protective effect of psychological resilience between male and female children.

The moderation effect of age was significant, with *β* = −0.082, SE = 0.038, and *p* < 0.05. Simple slope analysis showed that the pathway coefficient of children aged 8–9 years (*β* = 0.495, *p* < 0.001) was significantly higher than that of children aged 10–12 years (*β* = 0.381, *p* < 0.001).

The moderation effect of injury severity was significant, with *β* = −0.107, SE = 0.045, and *p* < 0.05. Simple slope analysis showed that the pathway coefficient of children with mild injuries (*β* = 0.518, *p* < 0.001) was significantly higher than that of children with moderate injuries (*β* = 0.402, *p* < 0.001) and severe injuries (*β* = 0.276, *p* < 0.01).

For the moderation effect of age: Simple slope analysis showed that the pathway coefficient for children aged 8–9 years was *β* = 0.495 (SE = 0.062, 95% CI = [0.373, 0.617], *p* < 0.001), while for children aged 10–12 years it was *β* = 0.381 (SE = 0.051, 95% CI = [0.281, 0.481], *p* < 0.001). For the moderation effect of injury severity: Simple slope analysis revealed coefficients of *β* = 0.518 (SE = 0.073, 95% CI = [0.375, 0.661], *p* < 0.001) for mild injuries, *β* = 0.402 (SE = 0.058, 95% CI = [0.288, 0.516], *p* < 0.001) for moderate injuries, and *β* = 0.276 (SE = 0.089, 95% CI = [0.102, 0.450], *p* < 0.01) for severe injuries. For the moderation effect of age: Simple slope analysis showed that the pathway coefficient for children aged 8–9 years was *β* = 0.495 (SE = 0.062, 95% CI = [0.373, 0.617], *p* < 0.001), while for children aged 10–12 years it was *β* = 0.381 (SE = 0.051, 95% CI = [0.281, 0.481], *p* < 0.001). For the moderation effect of injury severity: Simple slope analysis revealed coefficients of *β* = 0.518 (SE = 0.073, 95% CI = [0.375, 0.661], *p* < 0.001) for mild injuries, *β* = 0.402 (SE = 0.058, 95% CI = [0.288, 0.516], *p* < 0.001) for moderate injuries, and *β* = 0.276 (SE = 0.089, 95% CI = [0.102, 0.450], *p* < 0.01) for severe injuries.

## 4 Discussion

### 4.1 Direct protective effect of psychological resilience on sports injury recovery in local children

In this study, the results supported Hypothesis H1: Baseline psychological resilience significantly and positively predicted injury recovery at T1 and T2, with correlation coefficients of 0.407 and 0.462, respectively, (both *p* < 0.001). The direct effect accounted for 62.4% of the total effect.

This finding is consistent with the results of a previous study (Hu et al., 2025). Additionally, the study further found that the protective effect of psychological resilience was stronger in younger children (aged 8–9 years) and those with mild injuries. This may be because younger children rely more on external support from families and coaches, and the “sense of controllability over recovery” from mild injuries is more likely to be converted into rehabilitation motivation through the “goal focus” dimension of psychological resilience (Chen et al., 2021).

From a physiological mechanism perspective, children with high psychological resilience may reduce cortisol levels by regulating the function of the hypothalamic–pituitary–adrenal (HPA) axis—though this is an associative finding (not causal) due to cross-sectional physiological measurements. Data from T0 saliva sample tests in this study (*N* = 128) showed that the saliva cortisol level of the high psychological resilience group (CPRS total score ≥90, *n* = 65) was 1.35 ± 0.42 ng/mL 1 week after injury. This was 26% lower than that of the control group (CPRS total score <90, *n* = 63), which had a cortisol level of 2.28 ± 0.51 ng/mL (*t* = 9.87, *p* < 0.001). Excessively high cortisol levels can inhibit fibroblast proliferation, which affects ligament repair (Aizpurua-Perez et al., 2023). This provides a biological explanation for the protective effect of psychological resilience.

Meanwhile, after controlling for the “interval from injury to T0” in this study, the predictive effect of psychological resilience on injury recovery remained significant (*β* = 0.196, *p* < 0.001). This ruled out the confounding influence of “differences in the initial injury stage” on the results and further verified the direct protective effect of psychological resilience.

### 4.2 Differentiated mediating roles of emotional regulation in local children

The results supported Hypothesis H2: The two mediating pathways of emotional regulation showed opposite directions.

First was the positive mediation of cognitive reappraisal: Psychological resilience enhanced self-efficacy by improving cognitive reappraisal ability, with an indirect effect of 0.098. This is consistent with Gross’s model (Tang et al., 2022). Influenced by the local cultural context of “positive education,” children are more likely to reconstruct injury-related cognition through cognitive reappraisal. For example, they may view “being unable to attend PE class” as “an opportunity to learn rehabilitation knowledge”. This reduces negative emotions and strengthens rehabilitation beliefs.

Second was the negative mediation of expressive suppression: Psychological resilience improved self-efficacy by reducing expressive suppression, with an indirect effect of −0.029. Traditionally, local parents emphasize “endurance,” so some children are accustomed to suppressing pain-related emotions (Khodaparast et al., 2022). However, long-term expressive suppression can lead to increased cortisol levels (*β* = 0.201, *p* < 0.05), which weakens the formation of self-efficacy. It may also reduce access to family support—for instance, failing to report pain results in insufficient rehabilitation guidance.

Notably, the mediating effect of cognitive reappraisal (62.4%) was significantly higher than that of expressive suppression (18.5%). This suggests that cognitive reappraisal is a more suitable emotional regulation strategy for local children and can serve as a core target for interventions.

Based on existing research, pediatric resilience is associated with emotional adaptation (Chen et al., 2021), but no longitudinal studies have examined its relationship with sports injury recovery; while resilience enhances pain tolerance and rehabilitation compliance in adults (Ernst et al., 2022), developmental differences suggest it may operate via external support in children. Additionally, Gross (2002) model distinguishes between adaptive cognitive reappraisal and maladaptive expressive suppression in emotion regulation—cognitive reappraisal is linked to better coping in children (Aguinaga San José et al., 2023), whereas expressive suppression increases stress, and resilience is known to promote adaptive emotion regulation (Durish et al., 2019). Sports self-efficacy directly predicts rehabilitation engagement in children (Thorolfsson et al., 2023), and emotion regulation strategies shape self-beliefs (Xin et al., 2024); however, no longitudinal studies have tested the sequential pathway of “resilience → emotion regulation → self-efficacy → recovery” in children, with cross-sectional research only indicating correlations among these variables (Tamannaeifar and Suleimanian, 2024) and causal ordering and chain mediation remaining unvalidated. Therefore, this study hypothesizes that: baseline psychological resilience will be positively associated with injury recovery at T1 (3 months) and T2 (6 months); emotion regulation (cognitive reappraisal: positive; expressive suppression: negative) will mediate the relationship between psychological resilience and sports self-efficacy; sports self-efficacy will mediate the link between emotion regulation and injury recovery; and emotion regulation and sports self-efficacy will form a chain mediating effect between psychological resilience and injury recovery.

### 4.3 Core mediating role of sports self-efficacy

The results supported Hypotheses H3 and H4: Sports self-efficacy played a core mediating role between emotional regulation and injury recovery, and it formed a chain mediation with emotional regulation. The total mediating effect accounted for 37.9%.

This verifies Bandura’s social cognitive theory (Liu et al., 2025). Local children with high cognitive reappraisal ability enhance their self-efficacy through positive cognition. They are more willing to persist in rehabilitation training—such as completing 10 min of balance training every day—forming a “self-reinforcing cycle.” In contrast, children with high expressive suppression tend to experience “training avoidance” (e.g., skipping strength training) due to the accumulation of negative emotions, which delays recovery (Mousavi Nasab and Bahrami, 2022).

The single mediating effect of sports self-efficacy accounted for 21.7%, indicating that psychological resilience can directly improve self-efficacy. This may be because children with high psychological resilience are better at gaining efficacy from “small progress”—such as a 5° increase in knee flexion angle. In contrast, children with low psychological resilience tend to deny their abilities due to “mild pain after training” (Kim et al., 2023).

### 4.4 Limitations

Although the 128 samples from a single center met the requirements for mediating model testing, caution is needed when generalizing the results to other regions.

Regarding measurement tools: The Sports Self-Efficacy Scale used in this study is a revised version. It needs to be further verified by combining objective indicators, such as the check-in duration of rehabilitation training.

Social support variables were not included: Family support and coach support for local children may moderate the relationship between psychological resilience and emotional regulation, which needs to be supplemented in subsequent studies.

There is a lack of intervention research: Randomized controlled trials (RCTs) should be conducted to verify the effect of “cognitive reappraisal training” on local children.

This study still has some limitations. The longitudinal mediation design limits causal inference; bidirectional effects (e.g., recovery progress enhancing self-efficacy) cannot be ruled out. Questionnaire administration (online, guardian-assisted) may introduce response bias (e.g., guardians overreporting child’s recovery). The sample size (*N* = 128) is modest for complex SEM models, particularly moderation analyses, which may reduce statistical power to detect small effects.

## 5 Conclusion

Psychological resilience exerts a significant direct protective effect on sports injury recovery in children aged 8–12 years, and this effect is stronger in younger children (aged 8–9 years) and in children with mild injuries. Emotional regulation and sports self-efficacy form a chain mediation. The pathway “Psychological Resilience → Cognitive Reappraisal → Sports Self-Efficacy → Injury Recovery” is the main positive pathway, while the pathway “Psychological Resilience → Expressive Suppression → Sports Self-Efficacy → Injury Recovery” is the secondary negative pathway. Cognitive reappraisal may be a promising target for psychological interventions, though findings are limited to Harbin-based children and should not be generalized to other cultural contexts. Future research should use randomized controlled trials (RCTs) to test cognitive reappraisal training and include repeated measurements of all constructs to explore bidirectional relationships.

## Data Availability

The original contributions presented in the study are included in the article/supplementary material, further inquiries can be directed to the corresponding author/s.
